# Subtype-specific screen-detected and interval breast cancer rates in population screening: a BreastScreen Queensland cohort study

**DOI:** 10.1016/j.breast.2026.104938

**Published:** 2026-09-26

**Authors:** Naomi Noguchi, Nick Ormiston-Smith, Michael Luke Marinovich, Julie Moore, Nehmat Houssami

**Affiliations:** a School of Public Health, Faculty of Medicine and Health, University of Sydney, 301C Edward Ford Building, NSW, 2066, Australia; b BreastScreen Queensland, Clinical Support Queensland, Queensland Health, 15 Butterfield Street, Herston, QLD, 4006, Australia; c The Daffodil Centre, University of Sydney, and Cancer Council NSW, 1 King Street, Newtown, NSW, 2042, Australia; d Queensland Cancer Register, Cancer Alliance Queensland, Metro South Health, Burke Street Centre, 2 Burke Street, Woolloongabba, QLD, 4102, Australia

**Keywords:** Breast cancer, Population screening, Epidemiology

## Abstract

**Objectives:**

To quantify subtype-specific rates of screen-detected and interval breast cancers based on hormone receptor and HER-2 status in population-based breast cancer screening participants.

**Methods:**

Clinical and administrative data collected by BreastScreen Queensland (a biennial screening program) for consecutive mammography screens between 2017 and 2020 were analysed. Invasive cancer diagnosis and hormone receptors and HER-2 status were determined using BreastScreen Queensland Register and data linkage with the Queensland Cancer Register. Screen-detected and interval cancer rates for each subtype were determined overall and by age group.

**Results:**

Amongst 971,231 screening episodes in 557,110 women, 5635 screen-detected and 1702 interval cancers were diagnosed. Overall, cancer detection rate (CDR) at screening was 58.0 per 10,000 screens and interval cancer rate (ICR) was 17.5 per 10,000 screens. Hormone receptor (ER and/or PR) positive (HER-2 negative) CDR was 47.5 per 10,000, HER-2 positive (ER, PR any) CDR was 6.1 per 10,000, and triple-negative (ER, PR and HER-2 negative) CDR was 3.7 per 10,000 screens. Hormone receptor positive ICR was 12.0 per 10,000, HER-2 positive ICR was 2.5 per 10,000, and triple-negative ICR was 2.3 per 10,000 screens. Hormone receptor positive screen-detected cancers were particularly overrepresented in older age groups and overrepresentation of triple-negative interval cancers was constant across all age groups.

**Conclusions:**

To our knowledge, we report the world's first population-based estimates of both subtype-specific screen-detected and interval cancer rates respectively. These data provide a benchmark for investigating effects of new early detection strategies for breast cancer screening including those related to risk-based interventions.

## Highlights

• We report subtype-specific screen-detected and interval breast cancer rates.
• Subtypes were based on hormone receptors and HER-2 status.
• This provides a benchmark to assess effects of new detection strategies.
• Screen-detected cancers included a higher proportion of hormone receptor positive cancers.
• Triple-negative comprised a higher proportion of interval (vs screen-detected) cancers.

## Graphical abstract

**Figure:**
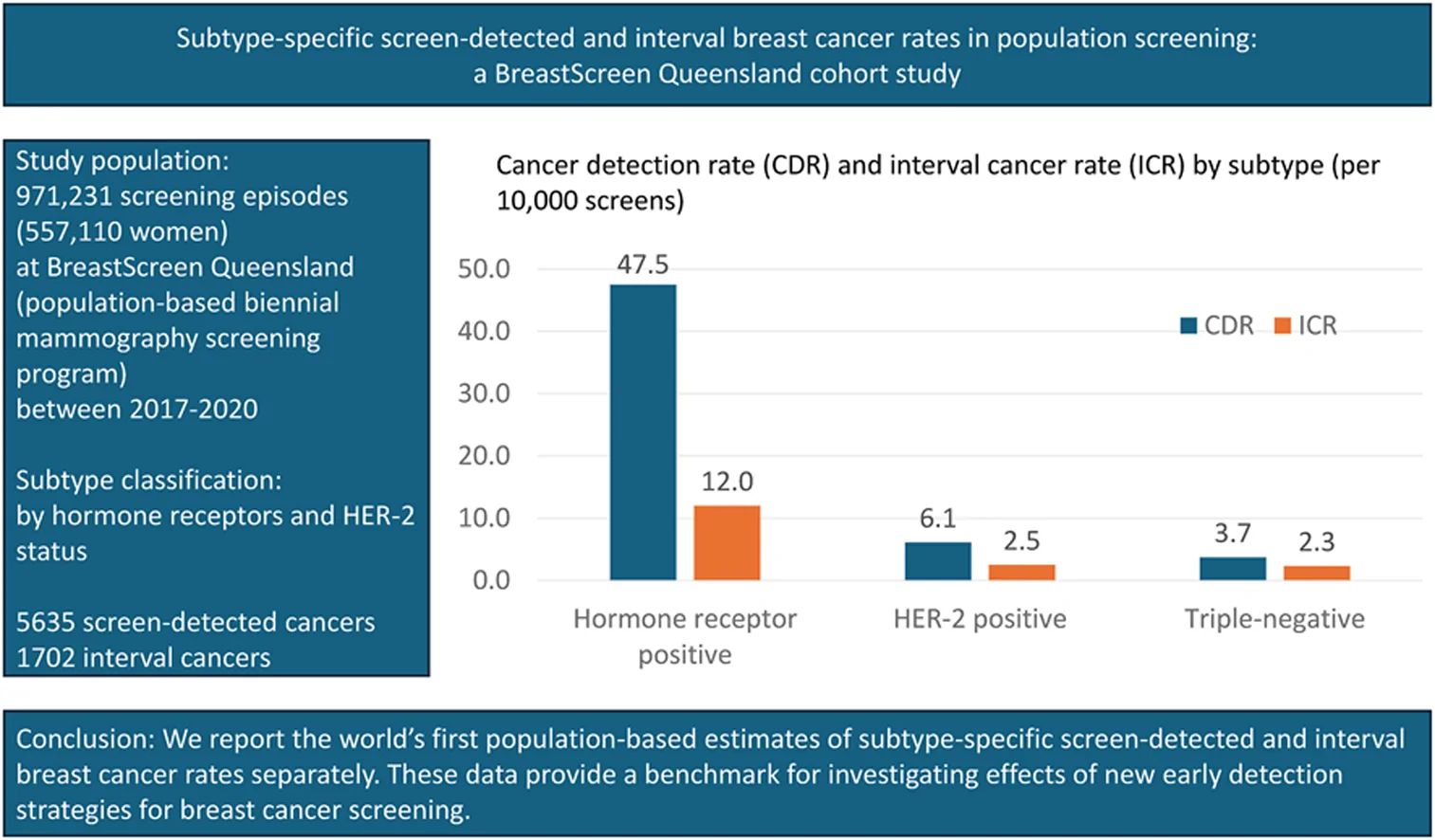

## 1 Introduction

Population-based breast cancer (BC) screening programs routinely monitor cancer detection rates and interval cancer rates, as these are initial and intermediate indicators, respectively, of screening effectiveness. To date, case-control studies have found that screen-detected BC include large proportions of hormone receptor (estrogen receptor (ER) and/or progesterone receptor (PR)) positive BCs whilst inter-screening interval BCs include a large proportion of triple-negative BCs [1,2]. This suggests that screening detects more BCs that respond to endocrine treatment but may be less effective at detecting triple-negative subtype that has limited treatment options and less favourable prognosis [3]. One of these studies examined if these characteristics varied between different age groups but their sample size was too small to observe trends [2].

These previous studies used a case-control design and compared proportions of each subtype between screen-detected and interval BCs. Although informative, case-control studies do not allow direct estimation of absolute population rates of each subtype. Using figures from case-control studies, absolute population rates can only be indirectly estimated by multiplying either screen-detected or interval cancer rates from other sources by the proportions of each subtype derived from case-control studies. Absolute population rates of each subtype are more relevant to policy-making because they tell us the size of the issue in a population and help us understand impacts of any policy changes regarding early detection of BC including those related to risk-stratified intervention [4].

This study aims to quantify subtype-specific rates of screen-detected and interval BCs based on hormone receptors and HER-2 status in a representative cohort of population-based screening participants to contribute to the knowledge base on early detection of BCs.

## 2 Materials and methods

### 2.1 Participants

Women aged 40 years and older are eligible for free mammograms every two years in the BreastScreen Queensland (BSQ) program. The target age group that is actively invited is 50 to 74. Women aged 40 to 49 and those aged ≥75 are not actively invited but are still able to have screening in the program if they wish. Women in the target age group who were deemed high-risk due to the following characteristics were recommended to screen annually: personal history of BC, first-degree female relative diagnosed with BC at <50 years, first-degree male relative diagnosed with BC at any age, and previous diagnosis of benign lesions associated with increased risk (atypical ductal hyperplasia, atypical lobular hyperplasia, and lobular carcinoma in-situ (LCIS) other than pleomorphic LCIS). Those with a first-degree family history of BC will have their screening interval changed to two years at 60 years of age providing no signs of BC have been found.

Consecutive screening participants between 1 January 2017 and 31 December 2020 were included in this study.

### 2.2 Measurement

This retrospective cohort study uses administrative and clinical data routinely collected by BSQ including interval cancer diagnosis through routine linkage to the Queensland Cancer Register.

The BSQ program routinely collects information on demographic characteristics, BC risk factors and breast symptoms using a self-administered registration form. In addition, details and results of all screening mammograms and assessments for recalled participants are recorded. Data on interval BCs including their hormone receptors and HER-2 status were obtained by linking to the Queensland Cancer Register which records all cancer diagnosis in the state to comply with legislation.

### 2.3 Outcomes

Primary outcomes were cancer detection rates (CDR) at screening and interval cancer rates (ICR) both per 10,000 screens by each hormone receptor (ER and PR) and HER-2 status and subtypes based on all three receptors.

Screen-detected cancers were defined as cancers detected following recall for assessment of screen-detected abnormalities. Interval BCs were defined as cancers in women whose screen results were negative and in whom BC diagnosis occurred before the next scheduled screen. If biennial screeners presented before 730 days (or annual screeners before 365 days) with a symptom and then were diagnosed with a cancer in the same breast, they were considered to have an interval BC.

‘Hormone receptor positive’ subtype was defined as (ER and/or PR positive) and (HER-2 negative). ‘HER-2 positive’ subtype could have any ER and PR status. ‘Triple negative’ subtype was defined as all of ER, PR and HER-2 negative. When there were multiple tumours or multiple foci in one tumour, receptor status was based on the tumour that was positive for each of ER, PR and HER-2 at individual level.

### 2.4 Statistical analysis

Characteristics of the cohort were summarized descriptively using percentages for categorical variables. 95% confidence intervals were computed using the binomial distribution.

Subtype-specific screen-detected and interval cancer rates per 10,000 screens were determined overall first, then stratified by age groups (40-49, 50-59, 60-69, and 70+) and compared with the chi-squared test. When the global test was statistically significant, post-hoc chi-squared tests were performed for each subtype and age group. The level chosen for statistical significance was p < 0.05.

Statistical analysis was performed using R version 4.5.1 [5] and SAS version 9.4 [6].

## 3 Results

Between 2017 and 2020, a total of 971,231 screens were performed in 557,110 women aged 40 years and older who attended BSQ: 5635 screen-detected and 1702 interval BCs were subsequently diagnosed.

Table 1 shows participant characteristics at the time of screening. The majority of the participants were in their fifties and sixties (34.7% were aged 50-59 and 32.2% were aged 60-69); 11.7% were first screens and the majority (65.8%) were re-screens from 15 to less than 27 months.

**Table 1:** Participant characteristics.

Characteristic		Screening episodes	
		N	%
**Age (years)**	40-49	161929	16.7%
	50-59	337133	34.7%
	60-69	312753	32.2%
	70 or older	159416	16.4%

**Socio-economic status (IRSD), quintile**			
	1st (most disadvantaged)	212474	21.9%
	2nd	187970	19.4%
	3rd	234061	24.1%
	4th	217763	22.4%
	5th (least disadvantaged)	115889	11.9%
	Unknown	3074	0.3%

**Time since previous screen (months)**			
	First screen	113922	11.7%
	Less than 15	72269	7.4%
	15 to less than 27	638910	65.8%
	27 or longer	146130	15.0%

**Personal history of breast cancer**			
	Yes	35808	3.7%
	No	935423	96.3%

**First degree family history of breast cancer**			
	Yes	122726	12.6%
	No	848505	87.4%

**Breast surgery including cosmetic surgery**^a^			
	Yes	146153	15.0%
	No	825078	85.0%

**Self-reported breast symptoms**			
	Yes	22795	2.3%
	No	948436	97.7%

IRSD = Index of Relative Socio-economic Disadvantage.

a ‘Cosmetic surgery’ refers to breast surgery for any condition and including breast reduction, augmentation or reconstructive surgery.

Table 2 shows screen-detected and interval cancer rates per 10,000 screens by hormone receptor and HER-2 status and subtypes based on these. At screening CDR was 58.0 per 10,000 screens; at follow-up ICR was 17.5 per 10,000 screens. The majority of screen-detected BCs were positive for ER and PR respectively whilst smaller proportions of interval BCs were hormone receptor positive. Either ER, PR or HER-2 status was missing for 70 (1.2%) screen-detected cancers and 66 (3.9%) interval cancers and therefore could not be classified into subtypes.

**Table 2:** Population rates of screen-detected and interval cancers by hormone receptors and HER-2 status and by subtypes (n = 971,231).

	Screen-detected cancers				Interval cancers			
	N	CDR	95% CI	%	N	ICR	95% CI	%
All cancers	5635	58.0	56.5-59.6		1702	17.5	16.7-18.4	

Estrogen receptor positive cancers	5028	51.8	50.4 -53.2		1349	13.9	13.2-14.7	
Estrogen receptor negative cancers	567	5.8	5.4 -6.3		330	3.4	3.1-3.8	
Estrogen receptor status missing	40	0.4	0.3-0.6		23	0.2	0.2-0.4	

Progesterone receptor positive cancers	4525	46.6	45.3 -48.0		1169	12.0	11.4-12.8	
Progesterone receptor negative cancers	1067	11.0	10.4 -11.7		506	5.2	4.8-5.7	
Progesterone receptor status missing	43	0.4	0.3-0.6		27	0.3	0.2-0.4	

HER-2 positive cancers	588	6.1	5.6-6.6		242	2.5	2.2-2.8	
HER-2 negative cancers	4981	51.3	49.9-52.7		1396	14.4	13.6-15.2	
HER-2 status missing	66	0.7	0.5-0.9		64	0.7	0.5-0.8	

Hormone receptor positive cancers^a^	4614	47.5	46.1-48.9	81.9	1168	12.0	11.2-12.7	68.6
HER-2 positive cancers^b^	588	6.1	5.6-6.6	10.4	241	2.5	2.2-2.8	14.2
Triple negative cancers^c^	363	3.7	3.4-4.1	6.4	227	2.3	2.1-2.7	13.3
Either receptor status missing	70	0.7	0.6-0.9	1.2	66	0.7	0.5-0.9	3.9

CDR = cancer detection rate at screening per 10,000 screens; ICR = interval cancer rate per 10,000 screens; 95% CI = 95% confidence interval.

a Hormone receptor positive cancers were defined as (ER and/or PR positive) and (HER-2 negative).

b HER-2 positive cancers could have any ER and PR.

c Triple negative cancers were defined as all of ER, PR and HER-2 negative.

Hormone receptor positive CDR was 47.5 per 10,000 screens, HER-2 positive CDR was 6.1 per 10,000 screens, and triple-negative CDR was 3.7 per 10,000 screens (81.9%, 10.4%, and 6.4% of screen-detected cancers respectively).

Hormone receptor positive ICR was 12.0 per 10,000 screens, HER-2 positive ICR was 2.5 per 10,000 screens, and triple-negative ICR was 2.3 per 10,000 screens (68.6%, 14.2%, and 13.3% of interval cancers respectively).

Proportions of subtypes differed between screen-detected BCs and interval BCs (p < 0.0001). Hormone receptor positive BCs were overrepresented in screen-detected BCs (81.9%) compared to interval BC (68.6%) (p < 0.0001). Triple-negative cancers were overrepresented in interval BCs (13.3%) compared to screen-detected BCs (6.4%) (p < 0.0001) and HER-2 positive cancers were also slightly overrepresented in interval BCs (14.2%) compared to screen-detected BCs (10.4%) (p = 0.006)).

Table 3 shows subtype-specific CDR and ICR by age groups. Proportions of subtypes differed between screen-detected BCs and interval BCs in all age groups (p < 0.0001 for women aged 40-49, 50-59 and 60-69, and p = 0.001 for those aged 70 years and older).

**Table 3:** Subgroup-specific screen-detected and interval cancer rates by age groups.

		Screen-detected cancers				Interval cancers			
		N	CDR	95% CI	%	N	ICR	95% CI	%
40-49 (n = 161929)	All cancers	539	33.3	30.6-36.2		300	18.5	16.6-20.7	
	Hormone receptor positive cancers^a^	426	26.3	23.9-28.9	79.0	223	13.8	12.1-15.7	74.3
	HER2 positive cancers^b^	77	4.8	3.8-5.9	14.3	31	1.9	1.4-2.7	10.3
	Triple negative cancers^c^	30	1.9	1.3-2.6	5.6	42	2.6	1.9-3.5	14.0
50-59 (n = 337133)	All cancers	1512	44.9	42.7-47.2		538	16.0	14.7-17.4	
	Hormone receptor positive cancers^a^	1193	35.4	33.4-37.5	78.9	342	10.1	9.1-11.3	63.6
	HER2 positive cancers^b^	205	6.1	5.3-7.0	13.6	110	3.3	2.7-3.9	20.4
	Triple negative cancers^c^	91	2.7	2.2-3.3	6.0	69	2.1	1.6-2.6	12.8
60-69 (n = 312,753)	All cancers	2073	66.3	63.5-69.2		536	17.1	15.8-18.7	
	Hormone receptor positive cancers^a^	1726	55.2	52.7-57.9	83.3	362	11.6	10.4-12.8	67.5
	HER2 positive cancers^b^	195	6.2	5.4-7.2	9.4	74	2.4	1.9-3.0	13.8
	Triple negative cancers^c^	132	4.2	3.6-5.0	6.4	74	2.4	1.9-3.0	13.8
70 or more (n = 159,416)	All cancers	1511	94.8	90.1-99.7		328	20.6	18.5-22.9	
	Hormone receptor positive cancers^a^	1269	79.6	75.4-84.1	84.0	241	15.1	13.3-17.2	73.5
	HER2 positive cancers^b^	111	7.0	5.8-8.4	7.3	26	1.6	1.1-2.4	7.9
	Triple negative cancers^c^	110	6.9	5.7-8.3	7.3	42	2.6	2.0-3.6	12.8

CDR = cancer detection rate at screening per 10,000 screens; ICR = interval cancer rate per 10,000 screens; 95% CI = 95% confidence interval.

a Hormone receptor positive cancers were defined as (ER and/or PR positive) and (HER-2 negative).

b HER-2 positive cancers could have any ER and PR.

c Triple negative cancers were defined as all of ER, PR and HER-2 negative.

Of screen-detected BCs, hormone receptor positive BCs were 79.0% (95%CI 75.6-82.5%) in women aged 40-49, 78.9% (95%CI 76.9-81.0%) in those aged 50-59, 83.3% (95%CI 81.7-84.9%) in those aged 60-69, and 84.0% (95%CI 82.1-85.8%) in those aged 70 years and older. Of interval BCs, they were 74.3% (95%CI 69.4-79.3%) in women aged 40-49, 63.6% (95%CI 59.5-67.6%) in those aged 50-59, 67.5% (95%CI 63.6-71.5%) in those aged 60-69, and 73.5% (95%CI 68.7-78.3%) in those aged 70 years and older. Hormone receptor positive screen-detected BCs were particularly overrepresented in older age groups and the difference was not significant in women aged 40-49 (p = 0.1 for women aged 40-49, p < 0.0001 for those aged 50-59, p < 0.0001 for those aged 60-69 and p = 0.002 for those aged 70 years and older).

Of screen-detected BCs, HER-2 positive BCs represented 14.3% (95%CI 11.3-17.2%) in women aged 40-49, 13.6% (95%CI 11.8-15.3%) in those aged 50-59, 9.4% (95%CI 8.2-10.7%) in those aged 60-69, and 7.3% (95%CI 6.0-8.7%) in those aged 70 years and older. Of interval BCs, they were 10.3% (95%CI 6.9-13.8%) in women aged 40-49, 20.4% (95%CI 17.0-23.9%)) in those aged 50-59, 13.8% (95%CI 10.9-16.7%) in those aged 60-69, and 7.9% (95%CI 5.0-10.9%) in those aged 70 years and older. Slight overrepresentation of HER-2 positive interval cancers was only seen in women aged 50-59 and 60-69 and there were no significant differences in those aged 40-49 and 70 years and older (p = 0.1 for women aged 40-49, p < 0.0001 for those aged 50-59, p = 0.001 for those aged 60-69 and p = 0.6 for those aged 70 years and older).

Of screen-detected BCs, triple-negative BCs constituted 5.6% (95%CI 3.6-7.5%) in women aged 40-49, 6.0% (95%CI 4.8-7.2%) in those aged 50-59, 6.4% (95%CI 5.3-7.4%) in those aged 60-69, and 7.3% (95%CI 6.0-8.6%) in those aged 70 years and older. Of interval BCs, they were 14.0% (95%CI 10.1-17.9%) in women aged 40-49, 12.8% (95%CI 10.0-15.7%) in those aged 50-59, 12.8% (95%CI 10.9-16.7%) in those aged 60-69, and 12.8% (95%CI 9.2-16.4%) in those aged 70 years and older. Overrepresentation of triple-negative interval BCs was constant across all age groups (p < 0.0001 for women aged 40-49, p < 0.0001 for those aged 50-59, p < 0.0001 for those aged 60-69 and p = 0.004 for those aged 70 years and older).

## 4 Discussion

BC is a heterogeneous disease as established by gene expression studies over the last two decades [7]. Subtypes of BC based on hormone receptors and HER-2 status are routinely used for prognostication and directing treatment strategies [8], but their use in early detection and prevention is limited.

As mammographic screening has been detecting more early-stage cancers that may be of indolent nature [9], calls have been made to detect and prevent more of the BCs that are likely to progress. For example, the European Collaborative on Personalized Early Detection and Prevention of Breast Cancer (ENVISION) Consensus Statement [4] has recommended that BC risk-assessment tools that will be used for risk-stratified BC screening should enable prediction of subtype-specific BC risk so women at higher risk of more aggressive subtypes can be targeted for prevention and early detection.

This study, to our knowledge, reports the world's first population-based estimates of subtype-specific screen-detected and interval BC rates, aligning the reporting of screening outcomes with the biological heterogeneity of BC. The subtype-specific BC rates that we have reported in this study provide a basis for subtype-specific BC risk prediction as well as providing a benchmark for monitoring effectiveness of risk-stratified interventions in the future.

Recently a study from the Netherlands also reported screen-detected BC rates by BC subtypes [10], but in our study, we also provide interval BC rates, with a suggestion that population BC screening programs could potentially report both outcomes (CDR and ICR) by BC subtype where feasible. Potentially, this may allow better understanding of the impact of changes in screening practice. Although there are some other studies that reported subtype-specific overall BC rates using European cancer registries [[11], [12], [13]], none of these report subtype-specific screen-detected and interval BC rates separately which we have done in this study. Thus, our study provides new information to inform screening practice.

In this study, hormone-receptor positive BCs were overrepresented in screen-detected BCs and triple negative BCs, and also HER-2 positive BCs to a lesser extent, were overrepresented in interval BCs. Our findings were consistent with the two case-control studies that compared proportions of subtypes between screen-detected and interval BCs as previously mentioned. These two studies both showed that screen-detected cancers included a larger proportion of hormone receptor positive cancers compared to interval cancers, and interval BCs included a larger proportion of triple negative cancers compared to screen-detected cancers [1,2]. This suggests that screening detects hormone receptor positive BCs effectively, but it is less able to detect triple-negative BCs early which tends to have higher ki67 proliferation index [14]. This may suggest that alternate strategies, such as risk-reducing intervention, may better target this BC subtype.

Bare et al. also investigated the difference in subtype distributions between age groups but their study lacked sample size to observe any age-related patterns. Our study showed that hormone-receptor-positive cancers were overrepresented in screen-detected BCs compared to interval BCs particularly in older age groups. This may be because hormone-receptor-positive cancers grow more rapidly in premenopausal women due to higher estrogen levels and are hence more likely to emerge in the inter-screen intervals. On the other hand, overrepresentation of triple-negative subtype in interval BCs compared to screen-detected BCs was constant across all age groups.

A limitation of our study is that we did not have data from genetic profiling that classifies molecular subtypes; we used receptor status based on conventional immunohistochemistry testing as these data are what is recorded in the BreastScreen Queensland and the Cancer Register. Nonetheless, this is a feasible approach for population BC screening programs that ascertain cancers and their prognostic information via cancer registries. Another limitation is inclusion of a small proportion of annual screeners (amongst those considered to have increased risk) in the Australian population screening context. This may have shifted the classification of some interval BCs into screen-detected BCs compared to a purely biennial screening setting.

## 5 Conclusion

We report new evidence comprising population-based estimates of subtype-specific screen-detected and interval BC rates; our findings show that hormone-receptor positive BCs were overrepresented in screen-detected BCs, while triple-negative BCs were overrepresented in interval (relative to screen-detected) BCs. Our findings were generally consistent with case-control studies. By providing absolute rates for screen-detected and interval BC from a large screened cohort, our results provide a benchmark for determining effects of new early detection strategies for BC including those related to risk-stratified screening. If new early detection strategies for BC are investigated or adopted, it will be relevant to understand if they improve earlier detection of HER-2 positive and triple-negative BCs.

## CRediT authorship contribution statement

**Naomi Noguchi:** Writing – review & editing, Writing – original draft, Software, Project administration, Methodology, Investigation, Conceptualization. **Nick Ormiston-Smith:** Writing – review & editing, Writing – original draft, Software, Investigation, Formal analysis, Data curation. **Michael Luke Marinovich:** Writing – review & editing, Software, Methodology, Investigation. **Julie Moore:** Writing – review & editing, Writing – original draft, Data curation. **Nehmat Houssami:** Writing – review & editing, Writing – original draft, Methodology, Investigation, Conceptualization.

## Ethics declaration

Written informed consent to take part in the study and to publish the article has been obtained from all participants or their legal representatives. The privacy rights of participants have been observed.

This study was performed in compliance with relevant laws, regulatory frameworks and guidelines where the research took place. This study was approved by the University of Sydney. (Approval No. 2025/HE000108)

## Data statement

Data availability is subject to approval by the data custodian, BreastScreen Queensland.

## Funding

The study was partly funded by NBCF Chair in Cancer Prevention grant (EC-21-001) awarded to NH.

## Declaration of Competing interest

The authors have no conflicts of interest to declare.

## References

[1] Barre M., Tora N., Salas D. (2015). Mammographic and clinical characteristics of different phenotypes of screen-detected and interval breast cancers in a nation-wide screening program. Breast Cancer Res Treat.

[2] Rayson D., Payne J.I., Abdolell M. (2011). Comparison of clinical-pathologic characteristics and outcome of true interval and screen-detected invasive breast cancer among participants of a Canadian breast screening program: a nested case-control study. Clin Breast Cancer.

[3] Anders C.K., Abramson V., Tan T., Dent R. (2016). The evolution of triple-negative breast cancer: from biology to novel therapeutics. Am Soc Clin Oncol Educ Book.

[4] Payashan N., Antonius A.C., Ivanus U. (2020). Personalized early detection and prevention of breast cancer: ENVISION consensus statement. Nat Rev Clin Oncol.

[5] R Core Team (2021). https://www.R-project.org/.

[6] SAS Institute Inc. Cary, NC, USA.

[7] Sorlie T., Perou C.M., Tibshirani R. (2001). Gene expression patterns of breast carcinomas distinguish tumor subclasses with clinical implications. Proc Natl Acad Sci USA.

[8] Goldhirsch A., Wood W.C., Coates A.S. (2011). Strategies for subtypes--dealing with the diversity of breast cancer: highlights of the St. Gallen international expert consensus on the primary therapy of early breast cancer 2011. Ann Oncol.

[9] Drukker C.A., Schmidt M.K., Rutgers E.J. (2014). Mammographic screening detects low-risk tumor biology breast cancers. Breast Cancer Res Treat.

[10] Lin Y., Sidorenkov G., Wang J. (2026). Impact of screening on late-stage breast cancer in Netherlands: a population-based cohort study (2007-2016). Breast.

[11] Hjerkind K.V., Johansson A.L.V., Trewin C.B., Russnes H.G., Ursin G. (2022 Jan 10). Incidence of breast cancer subtypes in immigrant and non-immigrant women in Norway. Breast Cancer Res.

[12] van Dooijeweert C., Deckers I.A.G., Baas I.O., van der Wall E., van Diest P.J. (2019 Jun). Hormone- and HER2-receptor assessment in 33,046 breast cancer patients: a nationwide comparison of positivity rates between pathology laboratories in the Netherlands. Breast Cancer Res Treat.

[13] Intrieri T., Manneschi G., Caldarella A. (2023 Jul). 10-year survival in female breast cancer patients according to ER, PR and HER2 expression: a cancer registry population-based analysis. J Cancer Res Clin Oncol.

[14] Hashmi A.A., Hashmi K.A., Irfan M., Khan S.M., Edhi M.M., Ali J.P., Hashmi S.K., Asif H., Faridi N., Khan A. (2019 Sep 23). Ki67 index in intrinsic breast cancer subtypes and its association with prognostic parameters. BMC Res Notes.

